# A DNA Vaccine Encoding the Gn Ectodomain of Rift Valley Fever Virus Protects Mice via a Humoral Response Decreased by DEC205 Targeting

**DOI:** 10.3389/fimmu.2019.00860

**Published:** 2019-04-25

**Authors:** Tiphany Chrun, Sandra Lacôte, Céline Urien, Charles-Adrien Richard, Matthias Tenbusch, Nicolas Aubrey, Coralie Pulido, Latifa Lakhdar, Philippe Marianneau, Isabelle Schwartz-Cornil

**Affiliations:** ^1^VIM-INRA-Université Paris-Saclay, Jouy-en-Josas, France; ^2^ANSES-Laboratoire de Lyon, Unité Virologie, Lyon, France; ^3^Institute of Clinical and Molecular Virology, University Hospital Erlangen, Friedrich-Alexander University Erlangen-Nürnberg, Medical Immunology Campus Erlangen, FAU Erlangen-Nürnberg, Erlangen, Germany; ^4^ISP, INRA, Université de Tours, UMR 1282 Team BioMAP, Nouzilly, France; ^5^ANSES-Laboratoire de Lyon, Plateforme d'Expérimentation Animale, Lyon, France

**Keywords:** Rift Valley fever virus, DNA vaccine, dendritic cell, DEC205, Gn glycoprotein

## Abstract

The Rift Valley fever virus (RVFV) is responsible for a serious mosquito-borne viral disease in humans and ruminants. The development of a new and safer vaccine is urgently needed due to the risk of introduction of this arbovirus into RVFV-free continents. We recently showed that a DNA vaccine encoding eGn, the ectodomain of the RVFV Gn glycoprotein, conferred a substantial protection in the sheep natural host and that the anti-eGn IgG levels correlated to protection. Addressing eGn to DEC205 reduced the protective efficacy while decreasing the antibody and increasing the IFNγ T cell responses in sheep. In order to get further insight into the involved mechanisms, we evaluated our eGn-encoding DNA vaccine strategy in the reference mouse species. A DNA vaccine encoding eGn induced full clinical protection in mice and the passive transfer of immune serum was protective. This further supports that antibodies, although non-neutralizing *in vitro*, are instrumental in the protection against RVFV. Addressing eGn to DEC205 was also detrimental to protection in mice, and in this species, both the antibody and the IFNγ T cell responses were strongly decreased. Conversely when using a plasmid encoding a different antigen, i.e., mCherry, DEC205 targeting promoted the antibody response. Altogether our results show that the outcome of targeting antigens to DEC205 depends on the species and on the fused antigen and is not favorable in the case of eGn. In addition, we bring evidences that eGn in itself is a pertinent antigen to be included in a DNA vaccine and that next developments should aim at promoting the anti-eGn antibody response.

## Introduction

Rift Valley Fever (RVF) is a mosquito-borne zoonotic viral disease that primarily affects ruminants. The etiologic agent Rift Valley Fever virus (RVFV) belongs to the *Phenuiviridae* family and is responsible for a high abortion rate in gravid females, a high mortality rate in newborns, and fetal deformities in domestic ruminants resulting in economic burden (1). Human infections with RVFV mainly occur through contact with body fluids or organs from infected livestock. In human, self-limiting febrile illness is generally observed and only a few infected individuals develop severe symptoms including blindness, encephalitis, hemorrhagic fever, and death. RVFV has spread outside its endemic areas of mainland Africa to Madagascar, the Comoros and the Arabian Peninsula, raising awareness of the risk of introduction and further dissemination of this pathogen into non-endemic continents where a large array of competent mosquitoes can transmit the virus. Commercial attenuated and inactivated veterinary vaccines are available in endemic countries. However, no safe and efficient vaccine for veterinary and human use is yet available in non-endemic countries (2).

Many RVFV candidate vaccines have been developed and among them, recombinant protein vaccines based on viral structural proteins showed promising results. Gn ectodomain (eGn) and Gc proteins confer protective immunity in the sheep natural host (3). Indeed, Gn and Gc are involved in virus attachment and fusion with the host cell respectively, and contain the virus neutralizing epitopes (4). Furthermore, an eGn subunit vaccine alone confers protection in sheep (5) and an eGn-based DNA vaccine confers partial protection in mice (6).

DNA vaccination is a promising strategy against emerging infectious diseases since it is safe, it can be manufactured in large scale and DNA vaccines are stable (7). Targeting antigen to selected surface receptors expressed on antigen presenting cells is an attractive strategy to improve DNA vaccine efficacy (8). Several studies reported the efficacy of antigen targeting to DEC205, a receptor expressed on DCs in many species including on sheep DCs (9, 10). Indeed, DNA vaccine strategies based on antigen targeting to DEC205 induced strong CD4^+^ and CD8^+^ T cell responses and enhanced the antibody (Ab) responses in mice (11–14), and even in cattle (15). Based on this context, we recently compared in lambs the efficacy of a DNA vaccine encoding eGn (peGn) and eGn fused to a single chain fragment variable (scFv) directed to the ovine DEC205 receptor (pscDEC-eGn) (16). These DNA vaccine-candidates were co-delivered by intradermal injection with a plasmid encoding GM-CSF as a genetic adjuvant (17), followed by surface electroporation (SEP) to enhance *in vivo* transfection (18). We have shown that peGn conferred a good clinical and viral protection in lambs and that pscDEC-eGn was less protective, it increased the IFNγ T cell responses and decreased the anti-eGn IgG responses.

The failure of DEC205 targeting to improve the anti-eGn IgG response in lambs is at odds with other reports showing the benefits of this targeting (11–14), yet in other species and with other antigens. This discrepancy may be due to the different expression profile or intracellular trafficking of DEC205 across species or, possibly, to the antigenic properties of eGn. In the present study, we evaluated the efficacy of peGn and pscDEC-eGn in the mouse reference model, and we sought to get further insight into the mechanisms underlying the protective immunity. Altogether our results show that the humoral response is protective and that DEC205 targeting of eGn is not favorable to this response in mice, like in sheep. However, we bring evidences that the DEC205 targeting outcome depends on the species, as the DEC205 targeting of eGn promoted the IFNγ T cell response in sheep and decreased this response in mice. Furthermore, we show that the DEC205 targeting of another antigen (mCherry) resulted in a mixed outcome, with an increase of the humoral response and a decrease of the IFNγ response, indicating that DEC205 targeting outcome also depends on the targeted antigen.

## Materials and Methods

### Cell Lines

Human embryonic kidney cells (HEK293) and African green monkey kidney cells (VeroE6) were maintained in culture with Dulbecco's Modified Eagle medium (DMEM, Life Technologies), supplemented with 5% decomplemented fetal bovine serum (FBS), and antibiotic solution at 37°C with 5% CO_2_. CHO_mDEC205_ (Chinese hamster ovary expressing murine DEC205) and CHO_neo_ (negative control) cell lines were maintained in DMEM supplemented with 5% FBS at 37°C with 5% CO_2_ without antibiotic solution.

### RVFV Strains

RVFV strains are classified into a single serotype and low genetic diversity among strains has been reported (19). The virulent strain RVFV ZH501, originally isolated from an infected patient (20), and the attenuated RVFV virus MP-12 strain (21) were provided by Michèle Bouloy (Paris Pasteur Institute). ZH501 strain was used in challenge experiment and the MP-12 strain was used in plaque reduction assay. The viruses were amplified on VeroE6 cells starting at a 0.01 MOI. The supernatant was harvested after 2 days, centrifuged for 10 min at 2,000 rpm and stored at −80°C until use.

### Production of Anti-RVFV Hyperimmune Mouse Ascitic Fluid

Anti-RVFV hyperimmune mouse ascitic fluid (HMAF) was obtained as previously described (16). Balb/c mice were inoculated intraperitoneally with inactivated RVFV-infected mouse brain suspensions in Freund's complete adjuvant on day 0 and 1 and in Freund's incomplete adjuvant on day 7 and 14. On day 21 and 28, mice were inoculated with virulent suspension in Freund's incomplete adjuvant. Finally, the mice were inoculated intraperitoneally with TG180 sarcoma cells on day 25. One week later, ascitic fluid was harvested and stored at −80°C until use.

### Construction and Production of Plasmids

A plasmid encoding Gn glycoprotein ectodomain of RVFV (peGn) was obtained as previously described (16). In order to generate targeted antigens to mouse DEC205, we used the published sequences of the variable region of the rat heavy (VH) and light (VL) chains of the anti-mouse DEC205 NLDC-145 clone, separated by a (G_4_S)_4_ flexible linker (22) and at its amino-terminus, we added a signal peptide derived from a murine IgG (22). A sequence encoding mCherry-6xHis-tag (pscDEC-mCherry) or eGn (pscDEC-eGn) was fused in frame to the carboxy terminus of the scFv anti-mouse DEC205 sequence with a second (G_4_S)_3_ linker and the final fusion proteins were cloned into a pCEP4 mammalian expression vector (Invitrogen). Plasmids encoding an irrelevant scFv fused to mCherry (pscCtrl-mCherry) or eGn (pscCtrl-eGn) were also similarly generated and cloned in the pCEP4 expression vector. The construction of these vectors encoding the scFv fusions was outsourced to Synogene, USA. The plasmid encoding murine GM-CSF (pGM-CSF) was purchased from InvivoGen (USA). A firefly luciferase expression plasmid (pLuc) was kindly provided by Stéphane Biacchesi (INRA, France). Plasmid productions for immunization were prepared using Endofree® Plasmid Giga Kits (Macherey-Nagel) and were stored at −20°C until use.

### Plasmids Expression Analysis in HEK293 Cells

Transfections with pscCtrl-mCherry and pscDEC-mCherry were performed in HEK293 cells and the supernatants containing scFv-mCherry proteins were purified by immobilized metal ion affinity chromatography, using IMAC Sepharose column charged with Ni^2+^, equilibrated with low-imidazole buffer (5 mM imidazole, 20 mM Tris-HCl (pH 7.4), 150 mM NaCl). After washing, the His-tagged proteins were eluted with high-imidazole buffer [800 mM Imidazole, 20 mM Tris-HCl (pH 7.4), 150 mM NaCl]. HEK293 cells were transiently transfected with pcDNA4 (negative control), peGn, pscCtrl-eGn, or pscDEC-eGn and the supernatants and cells were harvested at 72 h post-transfection. Cell pellets were lysed in lysis buffer (1% Triton X-100, 50 mM borate, 150 mM NaCl, pH 9) at a 1:9 (pellet:buffer volume) and sonicated. The supernatants of the HEK293 cells transfected with eGn encoding-plasmids were concentrated 20–40 times with Amicon® Ultra centrifugal filter 30 K (Millipore). Samples were resolved on a 12% SDS-PAGE under reducing conditions and blotted onto nitrocellulose. Blots were incubated overnight at 4°C with a polyclonal rabbit anti-dsRed [1:1,000 dilution, Clontech (catalog number 632496, USA)] followed by the horseradish peroxidase (HRP)-goat anti-rabbit (GAR) IgG (1:4,000 dilution, KPL, USA) or with anti-RVFV HMAF (1:1,000 dilution) followed by HRP-rabbit anti-mouse (RbAM) IgG (1:5,000 dilution, Sigma) in blocking buffer [PBS + 10 % fat-free milk (w/v)]. Immunoreactive bands were visualized with ECL substrate (Thermofisher). Image acquisitions were done with Chemidoc imaging system (Bio-rad).

### Staining and Flow Cytometry Analysis

CHO_mDEC205_ or CHO_neo_ cells were saturated on ice with RPMI supplemented with 4% horse serum for 30 min. To analyze the expression of DEC205, cells were incubated with an Alexa 647-conjugated (A647)-rat anti-mouse (RAM) DEC205 IgG2a (NLDC-145 clone, Ozyme), or control A647-RAM IgG2a (RTK4530 clone, Ozyme) during 30 min on ice. To analyze the binding of scCtrl-mCherry and scDEC-mCherry, the scFv-mCherry proteins were incubated with CHO cell lines on ice for 45 min. To analyze the binding of scDEC-eGn and scCtrl-eGn on CHO_mDEC205_ or CHO_neo_, the concentrated supernatants of HEK293 transfected cells with pcDNA4, peGn, pscCtrl-eGn, and pscDEC-eGn were incubated with CHO_mDEC205_ or CHO_neo_ cells for 2 h at 4°C. Cells were then incubated with anti-RVFV HMAF (1:1,000) and finally with A488-donkey anti-mouse (DAM) IgG (Jackson ImmunoResearch) (1:200). Dead cells were excluded by DAPI staining. Flow cytometry acquisitions were done with a LSR Fortessa flow cytometer (Becton Dickinson) and results were analyzed using FlowJo 10.0.6 software.

### Antigenic Sources for ELISA

Lysates from HEK293 cells transfected with peGn or with pcDNA4 were used as coating of ELISA wells to detect anti-eGn Abs and were prepared as described above. Purified mcherry protein produced in bacteria was used as coating to detect anti-mCherry Abs by ELISA. To produce mCherry, *E.coli* BL-21 were transformed with a plasmid expressing GST-TEV protease site fused to mCherry (pGEXTEV-4T3-mCherry). Protein expression was induced by adding 80 μg/ml of IPTG into LB medium with 100 μg/ml ampicillin at 28°C overnight. After solubilization of the pellet with a lysis buffer [Tris 50 mM (pH 7.8), NaCl 350 mM, DTT 2 mM, benzamidine 4 mM, Triton X-100 0.2% + lysozymes (10 mg/ml)], the recombinant protein was purified by binding to glutathione-sepharose 4B beads (GE Healthcare, Uppsala, Sweden). The tag was then removed by cleavage with TEV protease, and the mCherry protein released in the supernatant was collected and stored at −80°C until use.

### Optimization of Plasmid Transfection Efficacy in Mouse Skin

The experiment was approved by the COMETHEA ethic committee under the number 2015042719381737. Six to eight-week-old female Balb/c mice (Janvier, Le Genest, St Isle, France) were housed under BSL-1 in the animal facility (IERP, INRA, Jouy-en-Josas, France). In order to evaluate the V/cm parameter leading to optimal transfection in mouse skin, a pLuc reporter vector (50 μg) was intradermally injected in the left and right flanks. The injected skin areas were subjected to SEP using the CUY 21 EDIT system (BEX, Tokyo, Japan). Disk electrodes (3 mm) were loaded with conductive gel (Alcyon, France) and 6 electric pulses were applied during 10 ms with 90 ms interval with escalating pulses from 0 to 2,162 V/cm. Two days later, skin punch biopsies were harvested and lysed with 150 μl of lysis reagent (Luciferase Assay System E1500, Promega). Bioluminescence was measured using the *in vivo* Imaging System (IVIS-200, Xenogen, UK) after adding 100 μl luciferine substrate. The optimal V/cm was found to be 1,730 V/cm and was used in all subsequent experiments (Supplementary Material 1).

### Detection of eGn mRNA Expression by qRT-PCR in the Skin of Vaccinated Mice

The experiment was approved by the ComEth Anses/ENVA/UPEC ethic committee under the number 08/12/15-8. Ten Balb/c mice were injected intradermally with 100 μg peGn (3 mice), pscCtrl-eGn (3 mice), and pscDEC-eGn (4 mice) followed by SEP at 1,730 V/cm, as described above, 4 injection sites per mouse. The 4 injection sites per mouse (about 60 mg skin tissue in total) were homogenized by shaking with stainless steel bead (Thermo Fisher) in 1 ml of TRIzol reagent (Thermo Fisher Scientific) three times for 30 s using TissueLyserII (Qiagen). After centrifugation at 1,000 rpm for 5 min, total RNA purification were performed according to the manufacturer's instructions. The first RNA extract was purified using a RNeasy MinElute cleanup column. One μg purified RNA was treated twice with 5 μl of RNAse-free DNAse (30 Kunitz units, Qiagen) in order to remove any residual DNA plasmid from the injection (see below for control) and subjected to a final purification on column. Part of the RNA (100 ng) was reverse transcribed using the TaqMan® Reverse Transcription Reagents (Applied Biosystems). The cDNA (3 ng, duplicates) was subjected to a PCR reaction with the iTaq Univeral probes supermix (Biorad) for amplifying a eGn fragment with forward primer 5′-AGTGCGATGGGCAGTTGTC-3′, reverse primer 5′-TTCTTGAACACGGCAAATGG-3′ and a 6-FAM- ACAGCCCATGAGGTC-TAMRA probe (eGn codon-optimized sequences). The PCR cycling conditions were 95°C for 30 s, linked to 40 cycles of 95°C for 5 s and 60°C for 30 s. The non-reverse transcribed RNA (3 ng) of all samples was subjected to qPCR and no eGn PCR signal could be detected, showing that the transfected skin purified RNA did not include detectable residual plasmid. Non-transfected skin was used as negative control and did not generate any qPCR signal. The relative expression of eGn mRNA in transfected skin samples was calculated with the 2^−Δ*Ct*^ method using normalization with the GAPDH gene (forward primer 5′-GGGGTCGTTGATGGCAACA-3′, reverse primer 5′-AGGTCGGTGTGAACGGATTTG-3′), confirmed with the HPRT gene (forward primer 5′-CAGGCCAGACTTTGTTGGAT-3′, reverse primer 5′-TTGCGCTCATCTTAGGCTTT-3′), amplified using the iTaq UniverSYBR Green (Biorad). The lack of eGn qPCR signal detection was given a Cq of 40 for the calculation.

### Immunizations With eGn-Expressing Plasmids

The experiment was approved by the ComEth Anses/ENVA/UPEC ethic committee under the number 08/12/15-8. Six to eight-week-old female Balb/c mice (Janvier, Le Genest St Isle, France) were housed under BSL-3 conditions in the animal facility [Plateforme d'Expérimentation Animale (PFEA)-Anses in Lyon, France]. Mice were vaccinated under general anesthesia (isoflurane inhalation) two or three times at 3-weeks intervals, depending on experiments (3 in total), see figure legends. One hundred μg of peGn, pscCtrl-eGn or pscDEC-eGn were injected intradermally in the flank together with 20 μg of plasmid adjuvant (pGM-CSF) or without adjuvant, followed by SEP at 1,730 V/cm. A non-vaccinated control group (PBS with pGM-CSF) was included. Sera were collected at day 0, 21, 42, and 60 post-immunization and splenocytes were harvested at day 42 or 60 to analyze the T cell responses.

### Immunization With mCherry-Expressing Plasmids

The experiment was approved by the COMETHEA ethic committee under the number 2015042719381737. Six-week-old female Balb/c mice were housed under BSL-1 in the animal facility IERP, INRA, Jouy-en-Josas, France. The same immunization protocol as described above for immunization with eGn-expressing plasmids, was performed using pscCtrl-mCherry and pscDEC-mCherry with and without pGM-CSF. In these experiments, mice were vaccinated under general anesthesia (solution of ketamine and xylazine, 50 and 10 mg/kg respectively). Two experiments with pscCtrl-mCherry and pscDEC-mCherry with and without pGM-CSF were conducted and gave similar results.

### Evaluation of eGn- and mCherry-Specific T Cell Responses by ELISA

Spleens were collected 3 weeks after the last immunization and were mechanically dissociated through 100 μm nylon mesh strainers (BD). Splenocyte suspensions were treated with red blood cell lysis buffer (10 mM NaHCO_3_, 150 mM NaCl, 10 mM EDTA). Then 2 × 10^6^ cells were added to wells and re-stimulated with 5 μg/ml overlapping peptides (20 mers, offset 8, Mimotopes, Australia) in X-vivo medium (Ozyme, France) supplemented with 2% FBS and antibiotic solution at 37°C with 5% CO_2_ for 72 h. Overlapping peptides covering the N-terminal (AEDPHL […] LLPDSF) and C-terminal (VCFEHK […] NYQCHT) amino-acid sequences of the eGn protein (= 2 peptide pools) or overlapping peptides spanning the entire mCherry protein sequence (MVSKGE […] MDELYK) are listed in Supplementary Material 2. The overlapping peptides were divided in 2 pools initially diluted in H20:acetonitril (5 mg/ml) and distributed in 5 μl per well to have 1 μg of each peptide per well and avoid any toxicity of the H20:acetonitril diluent. An irrelevant peptide that derives from the HIV polymerase (IKDFHVYFRESRDALWKGPG) was used to estimate the non-specific responses (negative control). Cells cultured with ConA (25 μg/ml) were used as positive controls. All supernatants were assayed for IFNγ, IL-2, and IL-5 by ELISA (RSG ELISA kit, eBioscience). OD was measured at 450 nm using a TECAN microplate reader. The specific cytokine concentration was calculated with a standard curve using recombinant cytokines and obtained by subtracting OD 450 nm values from wells stimulated with the irrelevant peptide. In the case of stimulation with the 2 pools of eGn peptides, the specific cytokines concentrations obtained with each of the peptide pools were added to get the final total cytokine concentration.

### Evaluation of Anti-eGn and Anti-mCherry IgG Responses by ELISA

For anti-eGn IgG detection, 96-well-plates Nunc Maxisorp^TM^ (ThermoFisher) were coated overnight at 4°C either with 100 μl lysates from HEK293 cells transfected with peGn or with pcDNA4, diluted 1:500 in PBS + 0.01% sodium azide. Individual mouse sera were diluted 1:100 in PBS + 2 % fat-free milk (w/v) and incubated on the coated plates at 37°C for 1 h. The plates were subsequently incubated with HRP-RbAM IgG (Sigma) at a 1:4,000 dilution at 37°C for 1 h. HRP enzymatic activity was finally revealed using TMB substrate. OD was measured at 450 nm using a TECAN microplate reader. Corrected OD values were obtained by subtracting the signal of wells coated with lysates from HEK293 cell transfected with pcDNA4.

For anti-mCherry IgG detection, Half Area Clear Polystyrene High Bind Microplate 96-well plates (Corning®) were coated overnight at 4°C with 200 ng of recombinant mCherry protein produced as described above. Coated plates were washed in PBS 0.05% tween 20 (PBS-T) and saturated in diluent buffer (PBS-T + 5% FBS) for 1 h at 37°C. Individual mouse sera were serially diluted five-fold in PBS-T + 5% FBS starting at 1:100 and incubated on the coated plates at 37°C for 2 h. Antigen-bound Abs were revealed with an HRP-GAM IgG (KPL, USA) at a 1:4,000 dilution in diluent buffer at 37°C for 1 h. HRP enzymatic activity was finally revealed using TMB substrate. OD was measured at 450 nm using a TECAN microplate reader.

### Adoptive and Passive Transfer

The transfer experiment was approved by the ComEth Anses/ENVA/UPEC ethic committee under the number 11/10/16-7. Six to eight-week-old female Balb/c mice were immunized 3 times at 3-weeks interval with PBS or peGn with the pGM-CSF plasmid adjuvant combined with SEP as described above. Spleen and blood were collected 3 weeks after the last immunization. Splenocyte suspensions were obtained as above. Splenocytes from PBS or peGn-immunized mice were pooled and T cells were isolated by negative selection using a commercially available murine Pan T cell isolation kit (Miltenyi, USA) according to the manufacturer's instruction. The isolated cells included <1% of non-T cells as assessed by anti-CD3, CD4, and CD8 staining. Pooled sera of PBS or peGn-immunized mice were tested by ELISA before transfer (data not shown). Adoptive and passive transfers were performed with 6-week-old female Balb/c naïve mice. Three hundred microliters of serum or 10^7^ T cells in 200 μl of saline buffer with 2% horse serum were intraperitoneally injected per mouse and mice were challenged the day after.

### Challenge

The immunized mice were challenged 3 weeks after the last immunization with 10^3^ or 10^4^ pfu of the RVFV ZH501 strain in 100 μl volume by intraperitoneal route. After the challenge, weight, body temperature and clinical signs were daily monitored over 21 days. Brain and liver from moribund mice were extracted before being euthanized and were immediately stored at −80°C. At the end of the study, all animals were euthanized by cervical dislocation.

### RNA Extraction From Liver and Brain and Detection of Viral Gn RNA by qRT-PCR

Brain and liver were weighed and were homogenized in 500 μl of DMEM with stainless steel beads (ThermoFisher) 3 times for 30 s at 30 Hz using TissueLyserII. After centrifugation at 2,000 rpm for 5 min, supernatants were collected. One hundred μl of homogenate were used for RNA extraction. Total RNA was extracted with QIAMP viral RNA kit (QIAGEN) according to the manufacturer's instructions and eluted in 60 μl RNase-free water using QIAcube (QIAGEN). For quantification of viral RNA copies in organs from mice after challenge, a one-step qRT-PCR was performed using the SuperScript III Platinum One Step qRT-PCR kit (Invitrogen) as previously described (23). Primers were designed to amplify a nt 1,164–nt 1,258 Gn sequence of the M segment: forward primer 5′-AAA GGA ACA ATG GAC TCT GGT CA-3′, reverse primer 5′-CAC TTC TTA CTA CCA TGT CCT CCA AT-3′. A fluorescent probe was designed that hybridizes to the specific PCR product: FAM 5′-AAA GCT TTG ATA TCT CTC AGT GCC CCA A-3′ TAMRA. A standard corresponding to a region of the Gn RNA (nt 1,135 to nt 1,286 of the M segment) was synthetized using the Riboprobe® *in vitro* Transcription Systems (Promega). The qRT-PCR was performed with 3 μl of sample elution in 20 μl final mix and the cycling involved the following steps: reverse transcription at 45°C for 30 min, denaturation at 95°C for 5 min, amplification 45 cycles at 95°C for 5 s, and 57°C for 35 s. TaqMan run of experimental samples contained at least 2 replicates, a known positive control (RVFV ZH501 strain), a negative control (another Phlebovirus RNA), and nuclease-free water. The reactions were carried out in a LightCycler 480 (Roche). The number of viral RNA copies/ml in each sample was determined using the Gn RNA standard calibration curve. The results were expressed as the copy number of viral RNA per g of tissue.

### Virus Titration by Plaque Assay

The viral RNA-positive suspensions in qRT-PCR were serially diluted 1:10–1:10^6^ in DMEM medium and inoculated onto VeroE6 monolayer cells in 12-well-plates (5 × 10^5^ per well). One hour after adsorption at 37°C, 2 ml of diluted carboxymethylcellulose sodium salt with DMEM supplemented with 5% FBS (v/v) were added to each well and plates were incubated at 37°C in 5% CO_2_ for 5 days. Next, the plaque forming units (pfu) were revealed by 1% (w/v) crystal violet and the number of pfu/ml was calculated for each mouse serum.

### Plaque Reduction Assay

Neutralizing Ab before challenge were measured as previously described (16). Sera were diluted 1:10–1:160 in DMEM and incubated with 100 pfu of MP-12 virus at 37°C with 5% CO_2_ for 1 h. Next, the virus and the serum mix were added to VeroE6 cell monolayers which were further processed as described under the virus titration by plaque assay. The neutralizing Ab titers were established as the last dilution which inhibited 50% of the foci number per well compared to virus-only control titration.

### Statistical Analysis

Data were analyzed with the GraphPad Prism 6.0 software. As data did no pass a normality test (Kolmogorov-Smirnov), the unpaired non-parametric Mann-Whitney test was used to compare the T cell responses 2 by 2, the anti-eGn and anti-mCherry responses between different groups at the same time point. For anti-mCherry responses, a paired non-parametric Wilcoxon test was used to compare the Ab response at different time points vs. at day 0 within the same group. A non-parametric Kruskal Wallis test followed by Dunn's correction was used to compare the luciferase signals of transfected skin subjected to SEP vs. control skin.

## Results

### A Plasmid Encoding eGn Confers a Total Clinical Protection Against RVFV Infection in Mice

**Figure 1:**
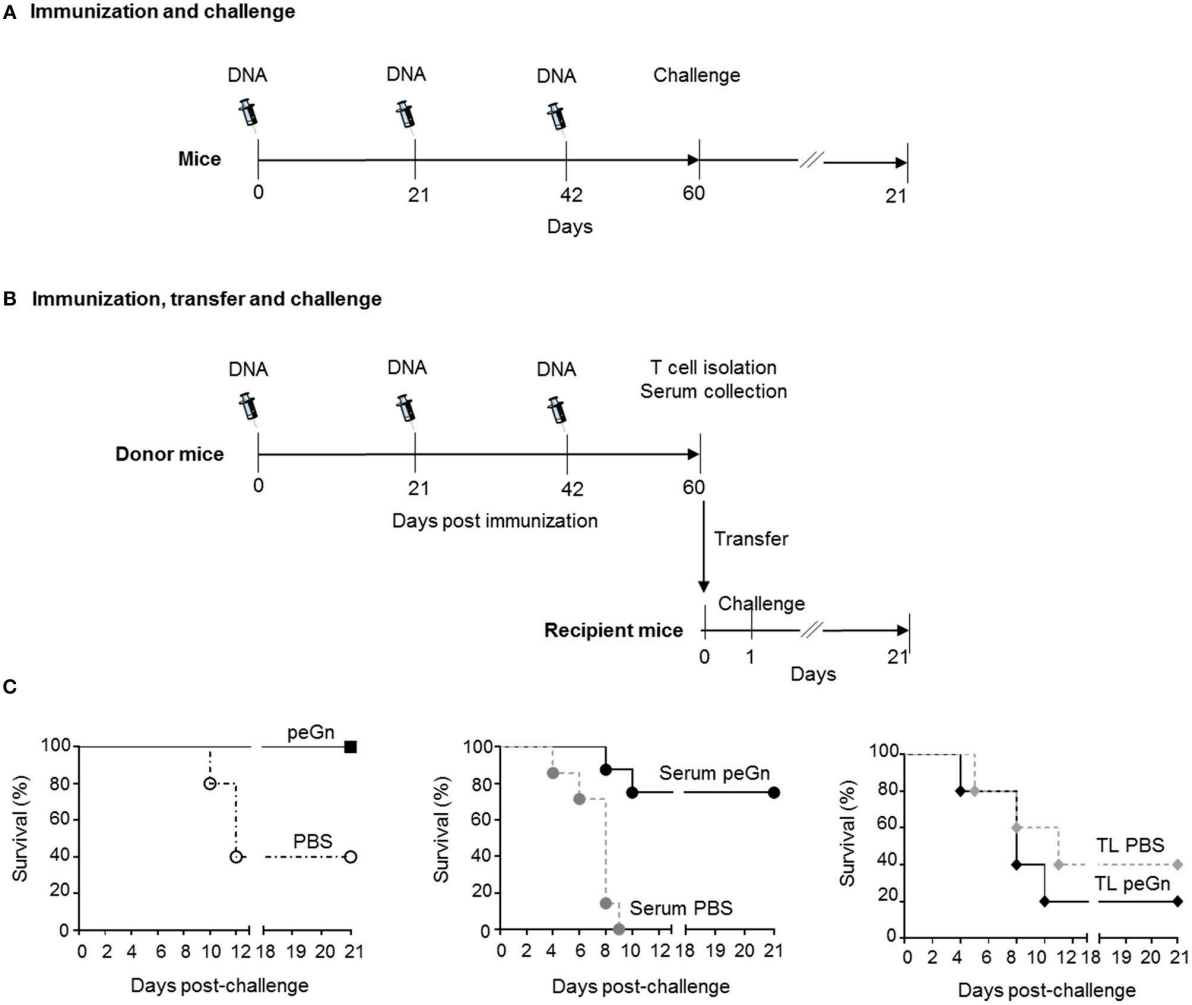
Effects of passive and adoptive transfers of immune effectors from peGn-vaccinated mice on the protection against RVFV. Six to eight-week-old female Balb/c were administered 3 times a day 0, 21, and 42 with 100 μg of peGn + 20 μg of pGM-CSF (immunized groups) or PBS + 20 μg of pGM-CSF (control groups) by intradermal injection in the flank and immediately followed by surface electroporation (1,730 V/cm). In **(A)** 3weeks after the last immunization (day 60), immunized and control mice (*n* = 5 per group) were challenged with 10^4^ pfu of RVFV (ZH501 strain). In **(B)** mice were euthanized to collect serum and splenocytes. Naïve recipient mice received 300 μl of serum or 10^7^ T cells from donor immunized or control mice. Twenty-four hours after the passive (serum) and adoptive (T cells) transfers, mice were challenged with 10^4^ pfu of RVFV ZH501, *n* = 5 for adoptive transfer and *n* = 7 for passive transfer. In **(C)**, the survival was monitored daily for 21 days in the vaccinated (peGn + pGM-CSF) and control (PBS + pGM-CSF) groups (left panel), in the passive transfer groups (middle panel) and in the adoptive transfer groups (right panel, TL stands for T lymphocytes) and the percentage of survival is shown in Table 1. The syringe drawing is from Servier Medical Art which provides open source illustrations.

We have previously shown that a DNA vaccine encoding eGn (peGn) administered with a plasmid encoding ovine GM-CSF was efficient at protecting lambs against RVFV, and that the anti-eGn IgG levels correlated with protection (16). To investigate whether this plasmid can also confer protection in mouse, Balb/c mice were immunized three times using the same delivery method than the one used in lambs, i.e., intradermal inoculation followed by surface electroporation [SEP (15), Figure 1A]. Plasmid encoding eGn or PBS combined with a genetic adjuvant encoding murine GM-CSF (pGM-CSF) were inoculated into the skin followed by SEP using the optimal electric parameters for plasmid expression in mouse skin (Supplementary Material 1). Immunized mice were subsequently challenged with 10^4^ pfu of the RVFV ZH501 strain and the survival was daily monitored during 21 days. Immunized mice with peGn + pGM-CSF were totally protected compared to control group (PBS + pGM-CSF) (Table 1, Figure 1C). No weight loss was measured (Table 1) and no apparent clinical signs were observed in the protected peGn-vaccinated mice. Conversely, 3 out of 5 mice in the control group became moribund, exhibited weight loss and died after infection. Moreover, viral RNA (ranging from 2.05 × 10^9^ to 2.28 × 10^9^ viral RNA copies/g) and infectious viral particles (ranging from 3.67 × 10^6^ to 2.60 × 10^7^ pfu/g) were found in the brain of 2 mice in this group (Table 1).

**Table 1:** Protection mediated by the T cells or sera from peGn-immunized mice upon RVFV challenge.

				**Liver**		**Brain**	
**Group**	***n*^**°**^ of mice surviving/total n^**°**^ of mice**	**DPC****^a^**	**% of initial weight****^b^**	**Viral RNA (RNA copy/g)**	**Viral titer (pfu/g)**	**Viral RNA (RNA copy/g)**	**Viral titer (pfu/g)**
Control mice (PBS + pGM-CSF)	2/5	9^I^	78.4	ND	ND	ND	ND
		12^I^	66.6	1,13E+06	–	2,28E+09	3,67E+06
		12^I^	86.4	1,37E+06	–	2,05E+09	2,60E+07
		22	99.1	–	–	–	−
		22	95.4	–	–	–	−
Immunized mice (peGn + pGM-CSF)	5/5	22	101.7	–	–	–	−
		22	98.3	–	–	–	−
		22	99.5	–	–	–	−
		22	100.4	–	–	–	−
		22	102.8	–	–	–	−
Transfer control T cells (PBS + pGM-CSF)	2/5	5^I^	96.2	ND	ND	ND	ND
		8^I^	70.1	6,21E+06	4,40E+02	7,72E+10	5,65E+07
		11^I^	64.1	2,98E+06	–	3,03E+10	1,94E+08
		22	105	–	–	2,13E+08	−
		22	102.6	–	–	4,71E+09	−
Transfer immunized T cells (peGn + pGM-CSF)	1/5	4^I^	90.6	ND	ND	ND	ND
		8^I^	69.2	1,36E+07	–	2,75E+13	3,84E+09
		8^I^	76.7	8,70E+06	–	9,58E+09	5,78E+07
		9^I^	81.5	–	–	8,31E+11	7,16E+08
		22	95.1	–	–	1,32E+09	−
Transfer control serum (PBS + pGM-CSF)	0/7	4^I^	91.2	6,50E+10	1,33E+06	1,98E+10	−
		6^I^	88.6	ND	ND	ND	ND
		8^I^	73.4	ND	ND	ND	ND
		8^I^	89.3	ND	ND	ND	ND
		8^I^	71.5	1,20E+07	1,11E+04	5,57E+12	1,25E+09
		8^I^	74.2	1,18E+07	–	4,86E+12	8,24E+08
		9^I^	72.2	1,45E+07	–	4,94E+10	3,27E+08
Transfer immunized serum (peGn + pGM-CSF)	5/7	8^I^	78.8	ND	ND	ND	ND
		10^I^	82.3	1,70E+06	–	9,02E+09	4,87E+07
		22	107.7	–	–	–	−
		22	101.5	–	–	2,39E+09	−
		22	107.3	–	–	–	−
		22	107.5	–	–	1,26E+09	−
		22	108.8	–	–	1,50E+09	−

*Mice were immunized, transferred, and challenged as described in Figure 1*.

a Death, days post challenge (DPC) either due to disease (I) or euthanized during the challenge protocol;

b *Values on the day before death; –, non-detected; ND, not done*.

### Passive Transfer of Serum From Pegn-Vaccinated Mice Protects Against RVFV Infection

To evaluate the role of humoral and cellular immunity mediated by peGn in this protection, we performed transfer experiments. Naïve recipient mice received 300 μl serum or 1 × 10^7^ purified T cells from mice immunized with peGn + pGM-CSF, or 300 μl serum or 1 × 10^7^ purified T cells from mice injected with pGM-CSF only. One day after passive and adoptive transfers, mice were challenged with 10^4^ pfu of RVFV (ZH501 strain). The survival and clinical signs were daily monitored for 21 days (Figure 1B). The majority of the mice that received the anti-eGn immune serum were protected (5/7 survival) and did not display clinical signs. Consistent with these results, no viral particles were found in the liver (Table 1). Viral RNA was found in brain but at much lower levels than in the mice injected with the control serum. Interestingly, all mice that received the control serum died (7/7 death) and exhibited severe clinical manifestations (hypothermia, significant weight loss, neurological manifestations, and ruffled fur). In this group, viral RNA (ranging from 1.18 × 10^7^ to 6.5 × 10^10^ viral RNA copies/g) and viral particles (ranging from 1.11 × 10^4^ to 1.33 × 10^6^ pfu/g) were found in liver, as well as in the brain (ranging from 1.98 × 10^10^ to 5.57 × 10^12^ viral RNA copies/g and from 3.27 × 10^8^ to 1.25 × 10^9^ pfu/g, Table 1). This high level of sensitivity in the mice receiving control serum might be explained by the well described immunosuppressive effect of IgG systemic injection (24). Recipient mice that received control T cells or T cells from immune mice were not protected and most of them succumbed upon infection, like the non-vaccinated mice did (Table 1). Mice in these two groups displayed clinical signs and became moribund and died upon the infection. Furthermore, viral RNA were found in the liver (ranging from 2.98 × 10^6^ to 1.36 × 10^7^ viral RNA copies/g), both viral RNA (ranging from 2.13 × 10^8^ to 2.75 × 10^13^ viral RNA copies/g), and viral particles (ranging from 5.65 × 10^7^ to 3.84 × 10^9^ pfu/g) were detected in brain of several mice in these groups (Table 1).

Altogether, this experiment showed that the transfer of humoral immune effectors from peGn-vaccinated mice protected mice against RVFV challenge contrary to the adoptively transferred T cells. This finding supports the role of anti-eGn immunoglobulins in the protection against RVFV.

### Generation and Expression of Plasmids Encoding DEC205-Specific scFv Fused to mCherry or eGn

**Figure 2:**
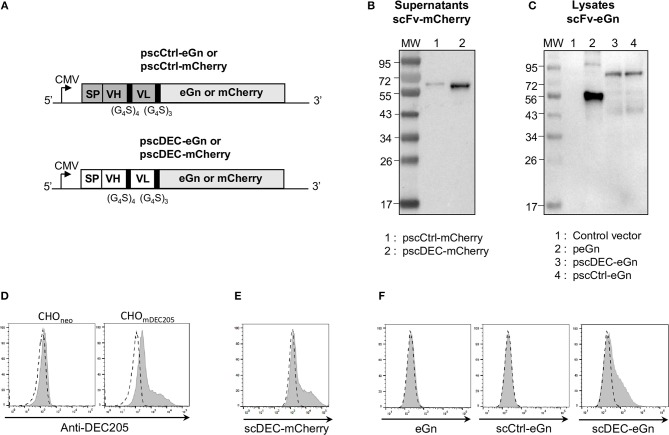
Design and characterization of the pscCtrl or pscDEC plasmids encoding the eGn and mCherry protein fusions with scFvs. **(A)** Schematic representation of pscCtrl and pscDEC fused with eGn or mCherry sequences. The pscCtrl (gray background) and pscDEC (white background) constructions include the signal peptide from VH of murine IgG (SP), the scFv (VH and VL) sequences, and the codon-optimized eGn or the mCherry sequences. The VH and VL sequences derived from the rat anti-DEC205 NLDC-145 mAb (scDEC) and from a control rat mAb (scCtrl) are connected together with a (G_4_S)_4_ linker and the scFv and antigen sequences are connected with a (G_4_S)_3_ linker. In **(B)** purified scCtrl-mCherry and scDEC-mCherry proteins collected from supernatants of transfected HEK293 cells were separated by SDS-PAGE under reducing conditions and mCherry was revealed with an anti-mCherry rabbit IgG followed by a HRP-GAR IgG. The predicted sizes of the expressed chimeric proteins are 57.6 and 57.5 kDa for the scDEC-mCherry and scCtrl-mCherry, respectively. In **(C)** HEK293 cells were transfected with pcDNA4 (control), peGn, pscCtrl-eGn, and pscDEC-eGn. The cell lysates were separated by SDS-PAGE under reducing conditions and eGn was revealed with an anti-RVFV HMAF followed by a HRP-GAM IgG. The predicted sizes of the recombinant proteins are 48.8 kDa for eGn, 78.8 kDa for the scCtrl-eGn, and 79 kDa for the scDEC-eGn. In **(D)** CHO cells expressing the murine DEC205 receptor (CHO_mDEC205_) or CHO_neo_ (control) were labeled with 10 μg/ml of A647-anti-DEC205 (gray) and compared to an A647-isotype control (dash line). In **(E)** CHO_mDEC205_ were labeled with 50 μg/ml of scCtrl-mCherry and scDEC-mCherry proteins from purified supernatants. The staining with scDEC-mCherry is depicted (gray) and compared to scCtrl-mCherry (dash line). In **(F)** CHO_mDEC205_ were labeled with the 20–40 times concentrated supernatants from the same number of transfected cells with peGn, pscCtrl-eGn, and pscDEC-eGn and eGn was revealed with the RVFV HMAF (1:1,000) followed with an A488-DAM IgG. The staining is depicted (gray) compared to a control supernatant (pcDNA4 empty vector, dash line). No signal was obtained with CHO_neo_ cells (not shown).

As said above, we have shown that DEC205 targeting improved the IFNγ T cell response in lambs but did not enhance the Ab response and protection against RVFV compared to a plasmid encoding eGn (16). As this finding contrasts with results from the literature obtained in other species, mainly in mice, and with other antigens, we aimed at evaluating the immunogenicity of DNA plasmids encoding DEC205-targeted eGn in mice as well as the immunogenicity of DNA plasmids encoding for another antigen, i.e., mCherry. We generated plasmids encoding murine DEC205 specific scFv fused to eGn (pscDEC-eGn) or mCherry (pscDEC-mCherry) as well as plasmids encoding an irrelevant scFv fused to these antigens (pscCtrl-eGn and pscCtrl-mCherry) as non-targeted controls (Figure 2A). The latter controls for the effect of molecular chimerisation of the antigens fused to a scFv, which was not evaluated in the sheep model (16). The plasmids were transiently transfected in HEK293 cells and supernatants were collected for assessing protein expression *in vitro*. Western blot analysis revealed a band corresponding to scCtrl-mCherry (theoretical size: 57.5 kDa for the chimera, mCherry being 28.8 kDa) and to scDEC-mCherry (57.6 kDa, theoretical size) (Figure 2B). As also reported in the case of scDEC-eGn in the sheep study (16), no bands could be detected by western blot even after concentration of the supernatants from HEK293 cells transfected with pscDEC-eGn and pscCtrl-eGn. The lack of detected signal can be due to the too low capacity of our anti-RVFV HMAF raised against the whole virus to detect the denaturated eGn chimeric molecules in Western Blot. Therefore, the expression levels of these plasmids were analyzed in the lysates of transfected cells where high quantity of proteins is expected to increase the efficacy of detection. Indeed as previously found in the sheep model (16), eGn alone was expressed at the expected size (48.8 kDa) (Figure 2C). Bands corresponding to scDEC-eGn (76.3 kDa) and scCtrl-eGn fusions (78.6 kDa) were observed at their expected predicted size and the eGn protein appeared to be more expressed than scDEC and scCtrl fused with eGn (Figure 2C).

### Binding of DEC205-Specific scFv Fused to mCherry or to RVFV eGn on the Murine DEC205 Receptor

In order to further assess the secretion of functional chimeric antigens, the supernatants of transfected HEK293 were evaluated for their binding capacity to CHO cells expressing murine DEC205 receptor (CHOm_DEC205_) vs. negative control CHO cells (CHO_neo_). The parental anti-DEC205 IgG labeled a fraction of CHOm_DEC205_ cells, whereas CHO_neo_ were not labeled, indicating that the stable CHO transfected cells indeed express the DEC205 molecule, although in a heterogeneous manner (Figure 2D). The scDEC-mCherry gave a similar staining pattern as the parental anti-DEC205 mAb (Figure 2E) showing the specific binding of scDEC on DEC205. Finally, the concentrated supernatant from HEK293 transfected cells with pcDNA4 (control), peGn, pscCtrl-eGn, and pscDEC-eGn were incubated with CHO_DEC205_. Similarly to scDEC-mCherry, scDEC-eGn stained the CHO_DEC205_ cells, whereas the eGn and scCtrl-eGn controls did not (Figure 2F).

In conclusion, pscDEC-eGn and pscDEC-mCherry express functional fusion proteins able to specifically bind the murine DEC205 receptor.

### Targeting eGn to DEC205 Decreased the Anti-eGn IgG Responses in DNA Vaccination in Mice

**Figure 3:**
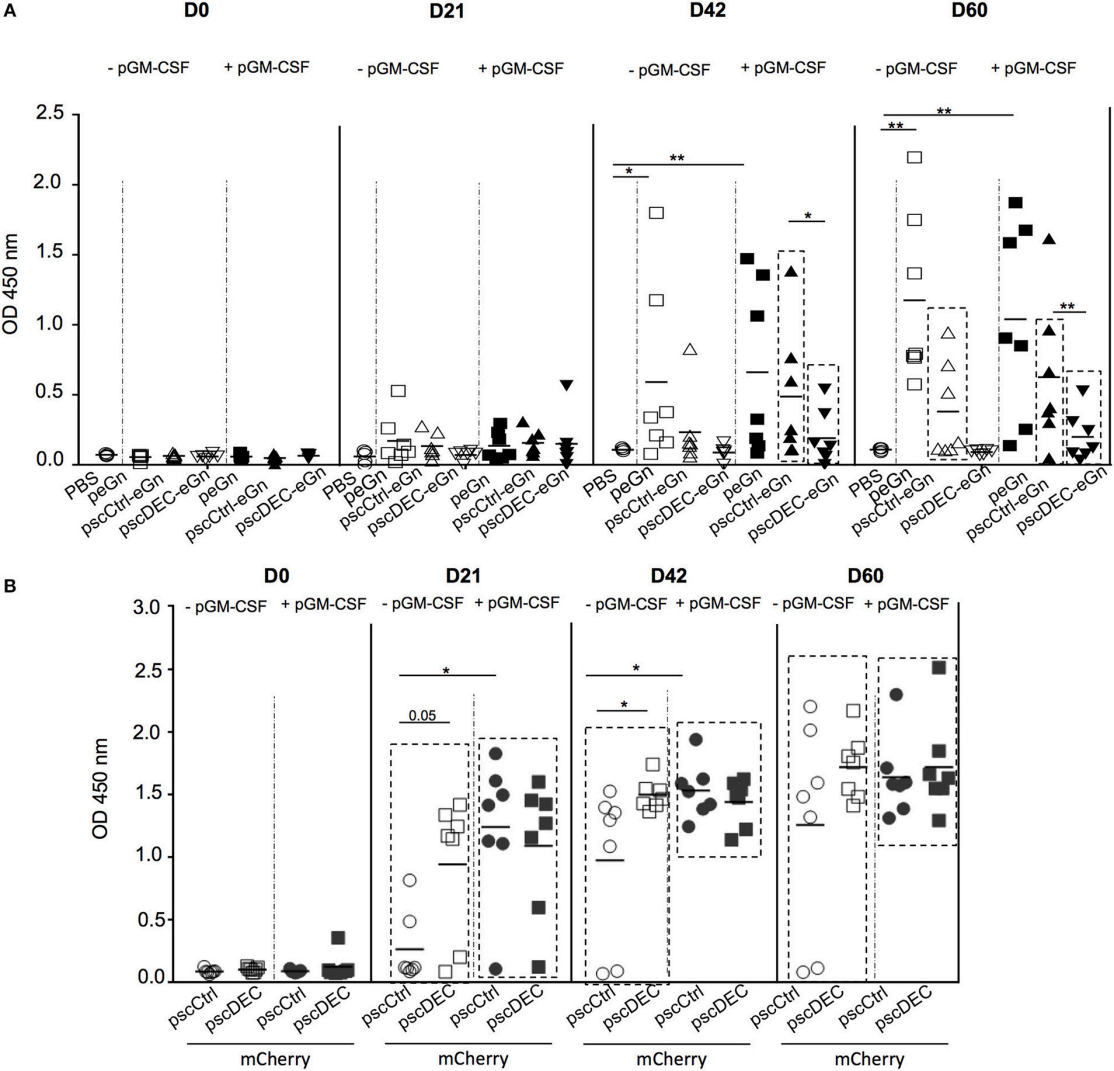
Ab responses induced by eGn and mCherry encoding plasmids. **(A)** Sera from pscDEC205-eGn, pscCtlr-eGn, and peGn (± pGM-CSF) immunized Balb/c (7 per group) were collected at day 0, 21, 42, 60, and were assayed for the detection of eGn-specific IgG using lysate from HEK293 cells transfected with peGn as coating. The OD signals at 450 nm from the individual sera collected at each time point and tested at a 1:100 dilution are shown. Each animal is represented with a distinct symbol and the mean OD value is indicated. *P-*values were determined using to the Mann-Whitney test (*p < 0.05; **p < 0.01). The significant difference between the OD values measured at each time point in the vaccinated vs. the PBS group is indicated by a box for clarity (*p* < 0.05). This experiment has been repeated twice with similar results. **(B)** Sera from pscDEC205-mCherry, pscCtlr-mCherry (± pGM-CSF) immunized Balb/c (7 per group) were collected at day 0, 21, 42, 60, and mCherry-specific IgG were evaluated in 1:100 diluted sera using recombinant mCherry as a coating antigen. Each animal is represented with a distinct symbol and the mean is indicated (*n* = 6–7 per group). The experiment has been repeated twice with similar results. *P*-values were determined using to the Mann–Whitney test (*p < 0.05; **p < 0.01). The significant differences between the OD values measured at day 0 in comparison to days 21, 42, and 60 are indicated by a box for clarity and were calculated using a non-parametric paired Wilcoxon test (*p* < 0.01).

In order to assess whether targeting eGn to DEC205 would promote the anti-eGn IgG response in mice whereas it did not in sheep, Balb/c mice were immunized with peGn, pscCtrl-eGn, pscDEC-eGn or PBS combined with or without pGM-CSF as described above. After 2 immunizations (D42), significant levels of anti-eGn IgG in serum was measured in peGn-immunized mice alone or with pGM-CSF (*p* < 0.05 vs. PBS), and pGM-CSF did not improve the Ab response (Figure 3A). At D42, a significant Ab response was detected in mice immunized with pscCtrl-eGn and pscDEC-eGn with pGM-CSF (*p* < 0.05 vs. PBS), and the level of anti-eGn IgG Ab was lower in the pscDEC-eGn that in pscCtrl-eGn immunized group. After 3 immunizations (D60), lower Ab responses were observed in the serum of mice immunized with pscDEC-eGn than in mice immunized with pscCtrl-eGn in combination or not with pGM-CSF (*p* < 0.01 and *p* < 0.05, respectively). At both time points, the levels of anti-eGn IgG appear to be higher in the peGn-vaccinated group than in the pscCtrl-eGn-vaccinated group (no statistically significant differences). This could be due to the effect of the chimerization. The ELISA-positive sera were tested for their capacity to neutralize RVFV in a reduction plaque assay and none of the sera were neutralizing at the difference with positive control sera from infected mice (data not shown).

Altogether, our results indicate that pscDEC-eGn was less efficient than pscCtrl-eGn and peGn for inducing anti-eGn IgG responses, independently of the co-administration of pGM-CSF, and that the anti-eGn IgG induced by our DNA vaccines were not able to neutralize RVFV *in vitro*. Thus, as similarly shown in lamb, DEC205 targeting of eGn is detrimental for the induction of anti-eGn Ab response.

### DEC205 Targeting of mCherry Promoted the Ab Responses in DNA Vaccination in Mice

In order to assess whether the effect of DEC205 targeting on the Ab response induction could be antigen-dependent, Balb/c mice were immunized with pscCtrl-mCherry or pscDEC-mCherry combined or not with pGM-CSF along with SEP. Significant mCherry-specific Abs were detected in immunized mice at all time point (*p* < 0.05 day 0 vs. day 21, 42, 60; Figure 3B). In absence of pGM-CSF, pscDEC-mCherry promoted a higher Ab response than pscCtrl-mCherry at day 21 and 42 (*p* = 0.05 and < 0.05, respectively). The plasmid adjuvant pGM-CSF promoted the Ab response induced by pscCtrl-mCherry, which was not further enhanced by DEC205 targeting. Thus, targeting eGn and mCherry to DEC205 have contrasted outcomes on the Ab responses, indicating that the effect of DEC205 targeting is antigen-dependent.

### DEC205 Targeting of eGn and mCherry Reduced the IFNγ and Not the IL-2 T Cell Responses in Mice

**Figure 4:**
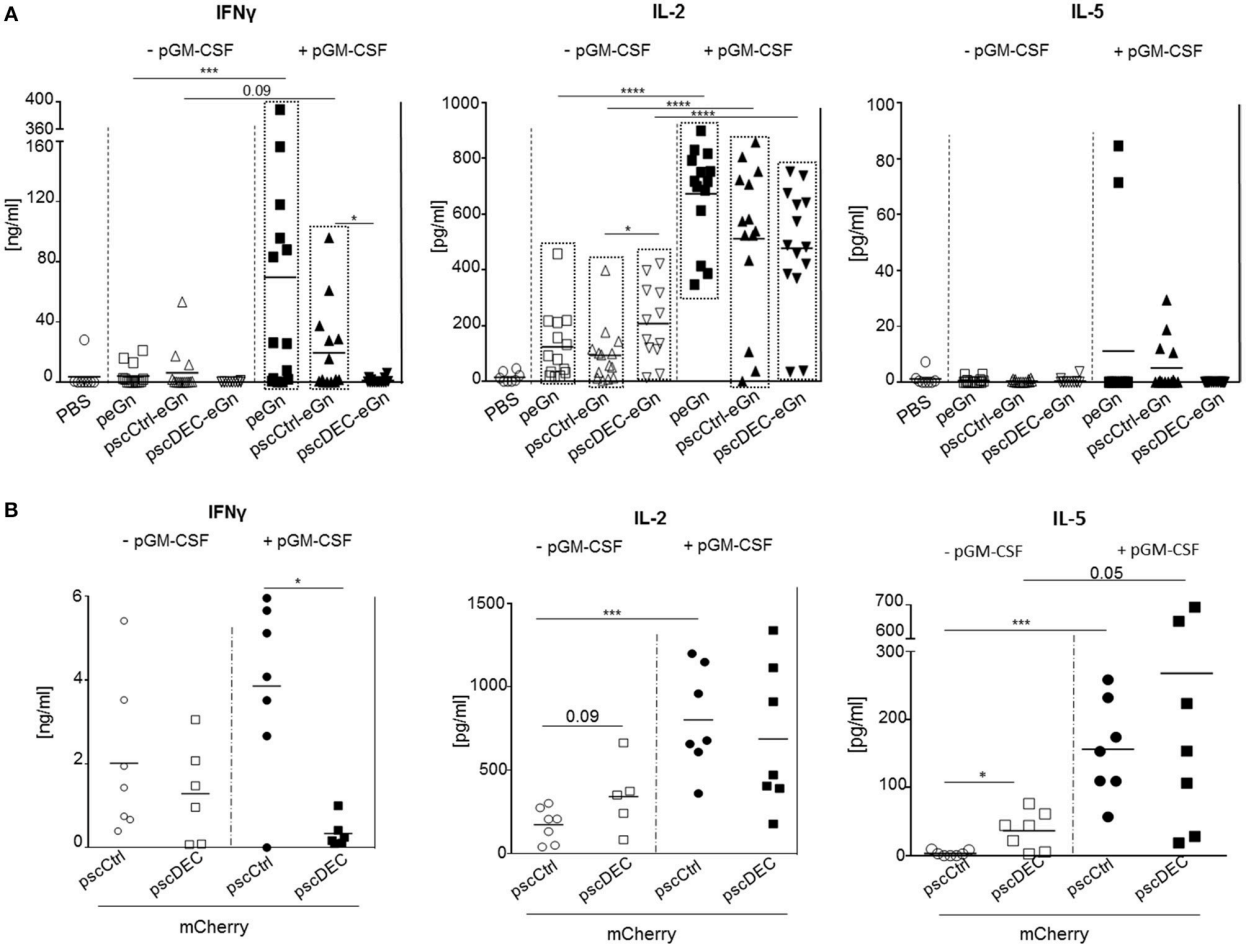
T cell responses induced by eGn and mCherry encoding plasmids. **(A)** The spleen cells were collected from mice (7 per groups) immunized with scDEC205-eGn, scCtlr-eGn, and eGn (± pGM-CSF), 3 weeks after the last immunization (day 60). Splenocytes (2 × 10^6^ cells per well) were re-stimulated *in vitro* with overlapping eGn peptides covering the N-terminal and C-terminal parts (5 μg/ml) or with an irrelevant peptide (5 μg/ml). IFNγ, IL-2, and IL-5 concentration were measured from cell culture supernatants at 72 h after the stimulation. The specific cytokine secretion was obtained using the signal from irrelevant peptide wells subtracted from conditions with specific eGn peptides covering the N-terminal or C-terminal parts. The total T cell responses per mouse was calculated by adding the cytokine concentrations obtained with the 2 peptide pools. Each symbol represents an individual animal and the mean is indicated (*n* = 5–7 per group). *P*-values were determined according to the Mann–Whitney test (*p < 0.05; ***p < 0.001; ****p < 0.0001). The significant differences between the cytokine levels of the PBS vs. the immunized groups is indicated by a box for clarity (*p* < 0.05). This experiment has been done twice with the same results. **(B)** Splenocytes (2 × 10^6^ cells per well) from the mice of the experiment described in this figure were collected at D60 and re-stimulated *in vitro* with overlapping mCherry peptides (5 μg/ml) or with an irrelevant peptide (5 μg/ml). IFNγ, IL-2, and IL-5 concentration were measured from cell culture supernatants at 72 h after the stimulation. The specific cytokine secretion was obtained from irrelevant peptides wells subtracted from condition with specific mCherry peptides. Each symbol represents an individual animal and the mean is indicated (*n* = 7 per group). This experiment with pGM-CSF has been done twice with the same results. *P-*values were determined according to the Mann–Whitney test (*p < 0.05; ***p < 0.001).

To analyze the eGn-specific T cell responses, total splenocytes were restimulated *in vitro* with 2 pools of overlapping peptides covering the sequence of eGn (N-ter and C-ter peptides) or with an irrelevant peptide as control. We attempted to investigate the production of IFN-γ by CD4^+^ and CD8^+^ T cells by intracellular cytokine staining but this method revealed to be not sensitive enough in our system. We then chose to measure the cytokine secretion in culture supernatants by ELISA (Figure 4A). Co-injection of pGM-CSF with peGn or pscCtrl-eGn was able to trigger significant IFNγ secretion (*p* < 0.05 vs. PBS) whereas pscDEC-eGn with pGM-CSF did not. All DNA vaccines were able to induce significant secretion of IL-2 in absence or in presence of pGM-CSF (*p* < 0.05 vs. PBS), and the response was higher when pGM-CSF was used (*p* < 0.001, DNA vaccines vs. DNA vaccines + pGM-CSF). Remarkably, the IL-2 secretion was even higher in mice immunized with pscDEC-eGn than pscCtrl-eGn without pGM-CSF (Figure 4A), indicating that eGn-targeted to DEC205 did not induce T cell anergy. Finally, no significant IL-5 secretion was measured in the supernatant of restimulated splenocytes from control and in all immunized groups (Figure 4A). In order to check that the reduced anti-eGn antibody and IFNγ T cell response obtained with pscDECeGn was not due to a defect of plasmid expression in mouse skin, we transfected peGn, pscCtrl-eGn, and pscDEC-eGn in mouse skin (3 or 4 mice per plasmid) and detected eGn using qRT-PCR, as done previously in the sheep study (16). The level of plasmid expression, which was detected in a fraction of the mice per group, was similar across plasmids (Supplementary Material 3). Therefore, we could not detect a defect in the *in vivo* expression of pscDECeGn as compared to the other plasmids, which could explain its altered immunogenicity.

We also analyzed the effect of DEC205 targeting of mCherry on the T cell responses in mice. The IFNγ T cell responses was lower in splenocytes from mice immunized with pscDEC-mCherry compared to pscCtrl-mCherry especially when pGM-CSF was used, and the IL-2 responses was similar in both groups, as in the case of eGn expressing vectors (Figure 4B). However, the IL-5 secretion was higher in the pscDEC-mCherry immunized group compared to the pscCtrl-mCherry immunized group, in line with the increased Ab response (*p* < 0.05 vs. pscCtrl-mCherry).

Altogether, our results indicate that while pscDEC-eGn and -mCherry were efficient at inducing IL-2 responses, they both reduced the IFNγ T cell responses compared to vectors encoding for the untargeted antigens. As higher IFNγ responses were obtained in sheep by targeting eGn to DEC205 (16), we can also conclude that the effect of DEC205 targeting on the IFNγ T cell response depends on the species. Thus, our immunogenicity results with the eGn and mCherry antigens indicate that the effects of DEC205 targeting in DNA vaccination depend on the fused antigen and on the species. This strategy was particularly inefficient in the case of eGn where protective immunity is mediated by the humoral IgG response.

### Targeting eGn to DEC205 Did Not Enhance Protection in Comparison to Non-targeted eGn

**Figure 5:**
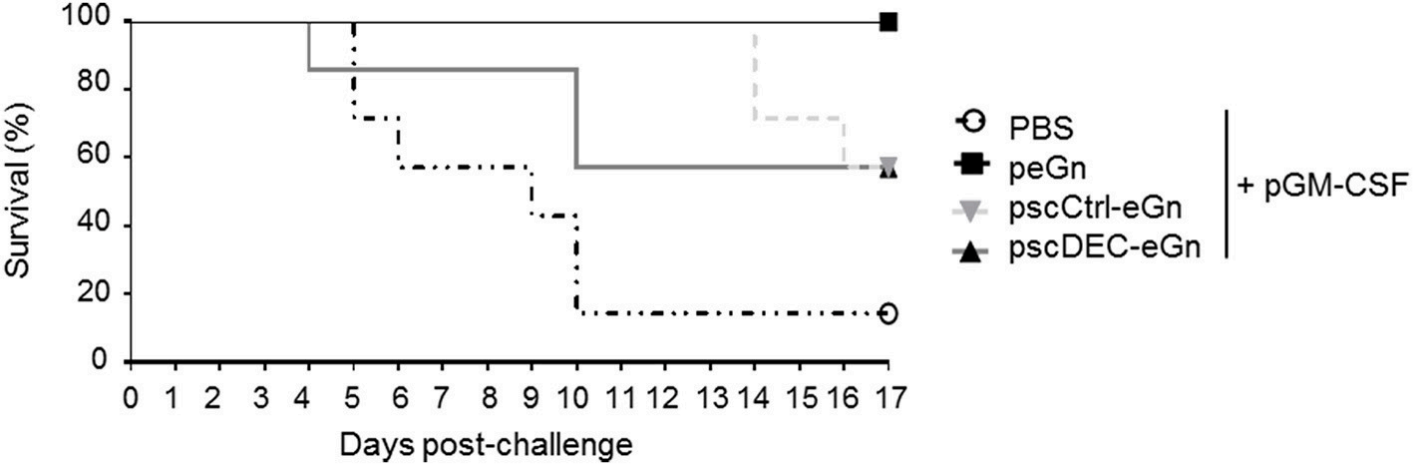
Protection induces by peGn, pscCtrl-eGn, and pscDEC-eGn against RVFV infection. Six to eight-week-old female Balb/c were immunized twice at day 0 and 21 with 100 μg of peGn, pscCtrl-eGn, pscDEC-eGn, or saline buffer (PBS) with 20 μg of pGM-CSF as an adjuvant. The plasmids were intradermally injected in the flank and immediately followed by surface electroporation (1,730 V/cm). Three weeks after the last immunization, mice were challenged with 10^3^ pfu of RVFV (strain ZH501). The survival was daily monitored for 17 days and the percentage of survival is shown (*n* = 7 per group).

We finally assessed the protection induced by scDEC-eGn, scCtrl-eGn and peGn vectors administered twice together with pGM-CSF. DNA-vaccinated mice were challenged with 1 × 10^3^ pfu of RVFV ZH501 strain. The survival was daily monitored during 17 days and the results are depicted in Figure 5. Five-days post-challenge, mice from control group start to succumbed upon the virulent ZH501 infection (6/7 death). All peGn vaccinated-mice survived contrary to non-vaccinated-mice as previously shown in Figure 1B. In line with the reduced immune responses, the pscDEC-eGn vaccinated mice were the first to die among all vaccinated mice (3/7 survival). The pscCtrl-eGn vaccinated mice were less well protected than the peGn vaccinated ones, possibly due to chimerization.

To conclude, our results indicate that in agreement with the reduction of immunogenicity and especially of the anti-eGn IgG levels, targeting eGn to DEC205 in mice has a negative effect on protection against RVFV. Altogether, peGn confers the best protection against RVFV, similarly to what was found in lambs (16).

## Discussion

In the present study, we showed that a DNA vaccine encoding eGn alone conferred a full protection against a RVFV in mice. To our knowledge, this is the first study reporting full clinical protection induced in mice with a DNA vaccine encoding eGn as only partial protection had been previously achieved with a eGn encoding plasmid and delivered by gene gun (6). Our results indicate that anti-eGn Abs are likely to be instrumental in protection while we could not show the implication of effector T cells by adoptive transfer. Interestingly, targeting eGn to DEC205 drastically reduced the capacity of eGn to induce IFNγ T cell and anti-eGn IgG responses in our mouse model. Our results show that the efficacy of DEC205 targeting on the different arms of the immune responses depends on the species, as well as on the vaccine antigen.

Neutralizing Abs are believed to be critical for the protection against RVFV (2). We showed by transfer experiments that humoral immune effectors, probably anti-eGn Abs, protected mice and favored viral clearance in the liver and the brain which are the main target organs of RVFV (25). The administration of purified immunoglobulins would be needed to fully demonstrate their role. However, it is unlikely that another component of the transferred serum from peGn + pGM-CSF-vaccinated mice would be implicated, also considering that the control serum which rather promoted to sensitivity to RVFV, was obtained from mice injected with pGM-CSF plasmid alone. Surprisingly the serum from peGn-vaccinated mice did not display any RVFV-neutralizing properties *in vitro*. The ability of Ab to exert viral neutralization is often related to the natural conformation of the vaccine antigen. In our case, expression of eGn by the plasmid vaccine *in vivo* may display differential post-translational modifications or folding than the subunit eGn protein vaccines that were able to induce neutralizing Ab in mice and sheep (5, 26). The eGn Abs induced by our DNA vaccine may also contribute to the viral clearance by other mechanisms such as complement-dependent inhibition or Ab-dependent cellular cytotoxicity (ADCC) or phagocytosis which remains to be investigated. In addition, it is possible that the classical *in vitro* neutralization assay does not faithfully recapitulate the *in vivo* neutralization and that the mouse sera were neutralizing *in vivo*. Finally, we have also shown that anti-eGn IgG levels correlated with protection in peGn-vaccinated lambs, and that these sheep IgG were also not neutralizing *in vitro* (16).

The targeting of vaccine antigen to DEC205 has been reported to be particularly efficient at promoting Th1 CD4^+^ T and CD8^+^ T cell responses, both in the case of protein vaccines (27, 28) and DNA vaccines (11–15). However, one of us reported that a DNA vector encoding influenza haemagglutinin targeted to DEC205 and delivered in the muscle by electroporation in mice led to a strong reduction of the IFNγ T cell response (29), as we also found here, both in the case of DNA encoding for DEC205-targeted eGn and mCherry antigens. In another study, we also reported that a DNA vector encoding for the Simian Immunodeficiency Virus p27 protein targeted to DEC205, delivered in muscle by electroporation, strongly reduced the IFNγ T cell response in monkey (30) whereas, as said above, we found that the IFNγ T cell response was enhanced by targeting eGn to DEC205 in sheep in DNA vaccination (16). The reasons for the discrepancies between all these studies are unclear and might reside in the ambivalent effect of DEC205 targeting which promotes effector T cell responses when the targeted DC are activated by adjuvants whereas it promotes regulatory T cell responses when the targeted DC are at steady state (27, 31). Electroporation induces an inflammatory response which was proposed to have adjuvant properties (32). However, in the case where plasmid expression is prolonged beyond the inflammatory response, the regulatory T cell response (Treg) may be the dominating response. Indeed influenza haemagglutinin targeted to DEC205 induced a potent Treg response in the mouse local lymph nodes (29). However, in our work, the total Foxp3^+^ T cell numbers in spleen and the secretion of IL-10 in eGn-restimulated splenocytes were not enhanced by the DEC205 targeting of eGn (data not shown), but we may have missed the Treg response if mainly located in the lymph nodes and/or if the response was antigen-specific. In any event, we also found that the reduction of the IFNγ responses induced by DEC205-targeted eGn and mCherry was particularly clear when pGM-CSF was co-injected. Whereas, pGM-CSF potently activated the T cell response induced by the plasmids encoding the non-targeted antigens, it may have induced the recruitment of immature dendritic cells (33), whose targeting via DEC205 would have further promoted a regulatory response.

DEC205 targeting of antigens has also been found efficient at promoting Ab responses, depending on the presence of adjuvant in the case of protein vaccines (34) and in the context of DNA vaccines in several instances (11–15). Interestingly however, we show here that this promotion can depend on the antigen fused to the scFv anti-DEC moiety, as an enhancement was found in the case of mCherry but a reduction in the case of eGn. We can speculate that these opposite outcomes may depend on the altered or maintained antigen conformation induced by the fusion with scFv, on the interactions of the antigens with other immune receptors together with the targeted receptor, on the kinetics of internalization of the fusion protein affecting the duration of the interaction with the B cell receptor, or on the presence of T cell epitopes favorable to the induction of T follicular and Th2 helper response (35). In favor of this later hypothesis, we found that pscDEC-mCherry and not pscDEC-eGn induced higher IL-5 responses than the plasmid encoding for the non-targeted antigens, and in addition pGM-CSF promoted the IL-5 response to mCherry and far less so to eGn.

The difference of outcome with DEC205 targeting of eGn between 2 different species regarding the IFNγ T cell response is puzzling. It is possible that the duration of plasmid expression was different between the two species, leading to target steady state DC in the case of mice and not in sheep. In addition, the routing of DEC205 may follow different maturation and compartment pathways and the effect of the combination with GM-CSF may induce different signaling.

Altogether we showed that a DNA vaccine encoding eGn is efficient at fully protecting mice from RVFV pathogenesis and we bring evidences that the Ab response is instrumental in this protection. Therefore, the improvement of DNA vaccine for application in target species should aim at inducing high anti-eGn Ab levels. We found that targeting eGn to DEC205 was not suitable for that purpose, both in mice and in sheep. Other DC receptors known to promote Ab response should be evaluated such as CLEC9A (36, 37). Our work also illustrates that the outcome of antigen-DC targeting remains unpredictable and at least depends on the antigen and the species. Knowledge in the mechanistic clues governing DC targeting efficacy is urgently needed to improve the success of this strategy.

## Ethics Statement

Animal studies were compliant with all the applicable provisions established by European directive 2010/63/UE. All the methods were performed by approved staff members in accordance with the relevant standard operating procedure approved by the mentioned ethics committee. All the animals used in this study were handled in strict accordance with good clinical practices and all efforts were made to minimize suffering.

## Author Contributions

TC performed most of the experiments, organized experiments, analyzed all results, and wrote the manuscript. IS-C designed and directed the study, coordinated the financial support and the experiments, wrote the request to the ethic committee (Jouy-en-Josas), performed experiments, guided the analyzes of the results, wrote sections, and directed and revised the writing of the manuscript. PM designed and directed the study, performed experiments, analyzed results and supervised the writing of the manuscript and the request to the ethic committee (Lyon). SL organized experiments, performed experiments, and help to write parts of the manuscript. CU performed experiments (immunizations, ELISA, and qRT-PCR). CP performed animal experiments. C-AR conducted scFv-mCherry and mCherry protein purification. LL contributed to animal experiments. MT participated in experiments with CHO cells transfected with DEC205 and in revision of the manuscript. NA participated to the design of the scFv anti-DEC205 and in revision of the manuscript.

### Conflict of Interest Statement

The authors declare that the research was conducted in the absence of any commercial or financial relationships that could be construed as a potential conflict of interest.

## References

[1] Mansfield KL Banyard AC McElhinney L Johnson N Horton DL Hernandez-Triana LM. Rift Valley fever virus: a review of diagnosis and vaccination, and implications for emergence in Europe. Vaccine. (2015) 33:5520–31. 10.1016/j.vaccine.2015.08.020 26296499

[2] Faburay B LaBeaud AD McVey DS Wilson WC Richt JA. Current Status of Rift Valley Fever Vaccine Development. Vaccines. (2017) 5:E29. 10.3390/vaccines5030029 28925970 PMC5620560

[3] Faburay B Wilson WC Gaudreault NN Davis AS Shivanna V Bawa B. A recombinant rift valley fever virus glycoprotein subunit vaccine confers full protection against rift valley fever challenge in Sheep. Sci Rep. (2016) 6:27719. 10.1038/srep27719 27296136 PMC4906348

[4] Besselaar TG Blackburn NK. The synergistic neutralization of Rift Valley fever virus by monoclonal antibodies to the envelope glycoproteins. Arch Virol. (1992) 125:239–50. 1642552 10.1007/BF01309641

[5] Kortekaas J Antonis AF Kant J Vloet RP Vogel A Oreshkova N. Efficacy of three candidate Rift Valley fever vaccines in sheep. Vaccine. (2012) 30:3423–9. 10.1016/j.vaccine.2012.03.027 22449427

[6] Bhardwaj N Heise MT Ross TM. Vaccination with DNA plasmids expressing Gn coupled to C3d or alphavirus replicons expressing gn protects mice against Rift Valley fever virus. PLoS Negl Trop Dis. (2010) 4:e725. 10.1371/journal.pntd.0000725 20582312 PMC2889828

[7] Jorritsma SHT Gowans EJ Grubor-Bauk B Wijesundara DK. Delivery methods to increase cellular uptake and immunogenicity of DNA vaccines. Vaccine. (2016) 34:5488–94. 10.1016/j.vaccine.2016.09.062 27742218

[8] Tregoning JS Kinnear E. Using plasmids as DNA vaccines for infectious diseases. Microbiol Spectr. (2014) 2:6. 10.1128/microbiolspec.PLAS-0028-2014 26104452

[9] Dutertre CA Wang LF Ginhoux F. Aligning bona fide dendritic cell populations across species. Cell Immunol. (2014) 291:3–10. 10.1016/j.cellimm.2014.08.006 25262488

[10] Contreras V Urien C Guiton R Alexandre Y Vu Manh TP Andrieu T. Existence of CD8α-like dendritic cells with a conserved functional specialization and a common molecular signature in distant mammalian species. J Immunol. (2010) 185:3313–25. 10.4049/jimmunol.1000824 20702727

[11] Nchinda G Kuroiwa J Oks M Trumpfheller C Park CG Huang Y. The efficacy of DNA vaccination is enhanced in mice by targeting the encoded protein to dendritic cells. J Clin Invest. (2008) 118:1427–36. 10.1172/JCI34224 18324335 PMC2263146

[12] Cao J Jin Y Li W Zhang B He Y Liu H. DNA vaccines targeting the encoded antigens to dendritic cells induce potent antitumor immunity in mice. BMC Immunol. (2013) 14:39. 10.1186/1471-2172-14-39 23941509 PMC3751307

[13] Hua Y Jiao YY Ma Y Peng XL Fu YH Zhang XJ. Enhanced humoral and CD8+ T cell immunity in mice vaccinated by DNA vaccine against human respiratory syncytial virus through targeting the encoded F protein to dendritic cells. Int Immunopharmacol. (2017) 46:62–9. 10.1016/j.intimp.2017.02.023 28259002

[14] Zaneti AB Yamamoto MM Sulczewski FB Almeida BDS Souza HFS Ferreira NS. Dendritic cell targeting using a DNA vaccine induces specific antibodies and CD4^+^ T Cells to the Dengue Virus Envelope Protein Domain III. Front Immunol. (2019) 10:59. 10.3389/fimmu.2019.00059 30761131 PMC6362411

[15] Njongmeta LM Bray J Davies CJ Davis WC Howard CJ Hope JC. CD205 antigen targeting combined with dendritic cell recruitment factors and antigen-linked CD40L activation primes and expands significant antigen-specific antibody and CD4(+) T cell responses following DNA vaccination of outbred animals. Vaccine. (2012) 30:1624−35. 10.1016/j.vaccine.2011.12.110 22240344

[16] Chrun T Lacote S Urien C Jouneau L Barc C Bouguyon E A Rift Valley fever virus Gn ectodomain-based DNA vaccine induces a partial protection not improved by APC targeting. NPJ Vaccines. (2018) 3:14 10.1038/s41541-018-0052-x 29707242 PMC5910381

[17] Saade F Petrovsky N. Technologies for enhanced efficacy of DNA vaccines. Expert Rev Vaccines. (2012) 11:189–209. 10.1586/erv.11.188 22309668 PMC3293989

[18] Gothelf A Gehl J. What you always needed to know about electroporation based DNA vaccines. Hum Vaccin Immunother. (2012) 8:1694–702. 10.4161/hv.22062 23111168 PMC3601144

[19] Bird BH Khristova ML Rollin PE Ksiazek TG Nichol ST. Complete genome analysis of 33 ecologically and biologically diverse Rift Valley fever virus strains reveals widespread virus movement and low genetic diversity due to recent common ancestry. J Virol. (2007) 81:2805–16. 10.1128/JVI.02095-06 17192303 PMC1865992

[20] Meegan JM. The Rift Valley fever epizootic in Egypt 1977-78. 1. Description of the epizzotic and virological studies. Trans R Soc Trop Med Hyg. (1979) 73:618–23. 538803 10.1016/0035-9203(79)90004-x

[21] Caplen H Peters CJ Bishop DH. Mutagen-directed attenuation of Rift Valley fever virus as a method for vaccine development. J Gen Virol. (1985) 66(Pt 10):2271–7. 10.1099/0022-1317-66-10-2271 4045430

[22] Coconi-Linares N Ortega-Davila E Lopez-Gonzalez M Garcia-Machorro J Garcia-Cordero J Steinman RM. Targeting of envelope domain III protein of DENV type 2 to DEC-205 receptor elicits neutralizing antibodies in mice. Vaccine. (2013) 31:2366–71. 10.1016/j.vaccine.2013.03.009 23499580

[23] Drosten C Gottig S Schilling S Asper M Panning M Schmitz H. Rapid detection and quantification of RNA of Ebola and Marburg viruses, Lassa virus, Crimean-Congo hemorrhagic fever virus, Rift Valley fever virus, dengue virus, and yellow fever virus by real-time reverse transcription-PCR. J Clin Microbiol. (2002) 40:2323–30. 10.1128/JCM.40.7.2323-2330.2002 12089242 PMC120575

[24] Prabagar MG Choi HJ Park JY Loh S Kang YS. Intravenous immunoglobulin-mediated immunosuppression and the development of an IVIG substitute. Clin Exp Med. (2014) 14:361–73. 10.1007/s10238-013-0255-4 23996469

[25] Gommet C Billecocq A Jouvion G Hasan M Zaverucha do Valle T Guillemot L. Tissue tropism and target cells of NSs-deleted rift valley fever virus in live immunodeficient mice. PLoS Negl Trop Dis. (2011) 5:e1421. 10.1371/journal.pntd.0001421 22163058 PMC3232203

[26] de Boer SM Kortekaas J Antonis AF Kant J van Oploo JL Rottier PJ. Rift Valley fever virus subunit vaccines confer complete protection against a lethal virus challenge. Vaccine. (2010) 28:2330–9. 10.1016/j.vaccine.2009.12.062 20056185

[27] Macri C Dumont C Johnston AP Mintern JD. Targeting dendritic cells: a promising strategy to improve vaccine effectiveness. Clin Transl Immunol. (2016) 5:e66. 10.1038/cti.2016.6 27217957 PMC4815026

[28] Lehmann CH Heger L Heidkamp GF Baranska A Luhr JJ Hoffmann A. Direct Delivery of Antigens to Dendritic Cells via Antibodies Specific for Endocytic Receptors as a Promising Strategy for Future Therapies. Vaccines. (2016) 4:8. 10.3390/vaccines4020008 27043640 PMC4931625

[29] Niezold T Storcksdieck Genannt Bonsmann M Maaske A Temchura V Heinecke V Hannaman D DNA vaccines encoding DEC205-targeted antigens: immunity or tolerance? Immunology. (2015) 145:519–33. 10.1111/imm.12467 25819746 PMC4515132

[30] Tenbusch M Ignatius R Nchinda G Trumpfheller C Salazar AM Topfer K. Immunogenicity of DNA vaccines encoding simian immunodeficiency virus antigen targeted to dendritic cells in rhesus macaques. PLoS ONE. (2012) 7:e39038. 10.1371/journal.pone.0039038 22720025 PMC3373620

[31] Mahnke K Ring S Enk AH. Antibody Targeting of “Steady-State” dendritic cells induces tolerance mediated by regulatory T Cells. Front Immunol. (2016) 7:63. 10.3389/fimmu.2016.00063 26941742 PMC4763042

[32] Todorova B Adam L Culina S Boisgard R Martinon F Cosma A. Electroporation as a vaccine delivery system and a natural adjuvant to intradermal administration of plasmid DNA in macaques. Sci Rep. (2017) 7:4122. 10.1038/s41598-017-04547-2 28646234 PMC5482824

[33] Matthews K Rhind SM Gossner AG Dalziel RG Hopkins J. The effect of gene gun-delivered pGM-CSF on the immunopathology of the vaccinated skin. Scand J Immunol. (2007) 65:298–307. 10.1111/j.1365-3083.2007.01902.x 17309785

[34] Cheong C Choi JH Vitale L He LZ Trumpfheller C Bozzacco L. Improved cellular and humoral immune responses *in vivo* following targeting of HIV Gag to dendritic cells within human anti-human DEC205 monoclonal antibody. Blood. (2010) 116:3828–38. 10.1182/blood-2010-06-288068 20668230 PMC2981538

[35] Amorim KN Rampazo EV Antonialli R Yamamoto MM Rodrigues MM Soares IS. The presence of T cell epitopes is important for induction of antibody responses against antigens directed to DEC205+ dendritic cells. Sci Rep. (2016) 6:39250. 10.1038/srep39250 28000705 PMC5175286

[36] Park HY Tan PS Kavishna R Ker A Lu J Chan CEZ. Enhancing vaccine antibody responses by targeting Clec9A on dendritic cells. NPJ Vaccines. (2017) 2:31. 10.1038/s41541-017-0033-5 29263886 PMC5674066

[37] Lahoud MH Ahmet F Kitsoulis S Wan SS Vremec D Lee CN. Targeting antigen to mouse dendritic cells via Clec9A Induces Potent CD4 T cell responses biased toward a follicular helper phenotype. J Immunol. (2011) 187:842–50. 10.4049/jimmunol.1101176 21677141

