# Supplementary material for: A DNA Vaccine Encoding the Gn Ectodomain of Rift Valley Fever Virus Protects Mice via a Humoral Response Decreased by DEC205 Targeting
Source: Front Immunol. 2019 Apr 25;10:860. doi: 10.3389/fimmu.2019.00860 (PMC6494931; doi:10.3389/fimmu.2019.00860)
Supplement: Supplementary file 1 [file Data_Sheet_1.docx]

**Supplementary material 1**


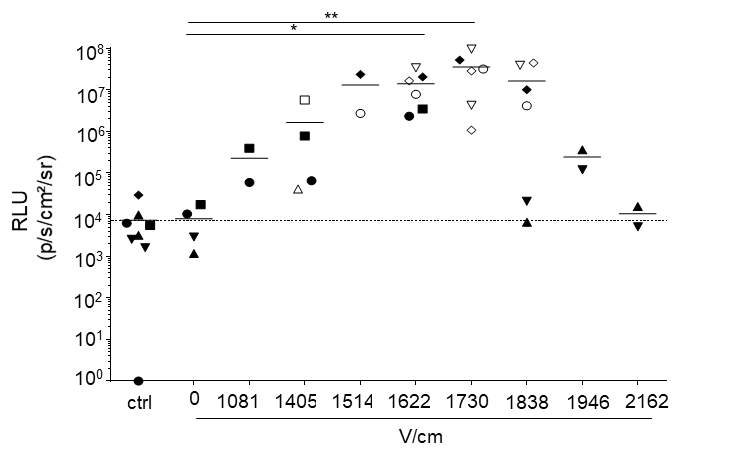


**Optimization of the SEP V/cm parameter for pLuc expression in mouse skin *in vivo.***

pLuc (50 µg) was intradermally injected in mouse skin followed by SEP (6 pulses, 10 ms per pulse with 90 ms intervals). Different voltages per cm pulses were applied. After 48 h, the site of injfection was harvested and processed to measure the luciferase activity (p/s/cm²/sr). The background signal is given by the non-treated skin (ctrl). The luciferase activity of the skin biopsies injected with pLuc and subjected to different V/cm from a given mice is represented by the same symbol. The luciferase activity mean obtained at a given V/cm from different mice is shown as a horizontal bar. P values were determined according to the Kruskal Wallis test with Dunn’s correction (*, p<0.05; **, p <0.01).
