# Supplementary material for: A DNA Vaccine Encoding the Gn Ectodomain of Rift Valley Fever Virus Protects Mice via a Humoral Response Decreased by DEC205 Targeting
Source: Front Immunol. 2019 Apr 25;10:860. doi: 10.3389/fimmu.2019.00860 (PMC6494931; doi:10.3389/fimmu.2019.00860)
Supplement: Supplementary file 2 [file Data_Sheet_2.docx]

Supplementary material 2: overlapping psetides for specific T cell restimulation of the eGn and mCherry proteins

| eGn protein | | mCherry |
| --- | --- | --- |
| Pool eGn1 | Pool eGn2 | Pool mCherry |
| AEDPHLRNRPGKGHNYIDGM | LKTEENLLPDSFVCFEHKGQ | MVSKGEEDNMAIIKEFMRFK |
| RPGKGHNYIDGMTQEDATCK | PDSFVCFEHKGQYKGTMDPG | NMAIIKEFMRFKVHMEGSVN |
| IDGMTQEDATCKPVTYAGAC | HKGQYKGTMDPGQTKRELKS | MRFKVHMEGSVNGHEFEIEG |
| ATCKPVTYAGACSSFDVLLE | MDPGQTKRELKSFDISQCPK | GSVNGHEFEIEGEGEGRPYE |
| AGACSSFDVLLEKGKFPLFQ | ELKSFDISQCPKIGGHGSKK | EIEGEGEGRPYEGTQTAKLK |
| VLLEKGKFPLFQSYAHHRTL | QCPKIGGHGSKKCTGDAAFC | RPYEGTQTAKLKVTKGGPLP |
| PLFQSYAHHRTLLEAVHDTI | GSKKCTGDAAFCSAYECTAQ | AKLKVTKGGPLPFAWDILSP |
| HRTLLEAVHDTIIAKADPPS | AAFCSAYECTAQYANAYCSH | GPLPFAWDILSPQFMYGSKA |
| HDTIIAKADPPSCDLQSAHG | CTAQYANAYCSHANGSGIVQ | ILSPQFMYGSKAYVKHPADI |
| DPPSCDLQSAHGNPCMKEKL | YCSHANGSGIVQIQVSGVWK | GSKAYVKHPADIPDYLKLSF |
| SAHGNPCMKEKLVMKTHCPN | GIVQIQVSGVWKKPLCVGYE | PADIPDYLKLSFPEGFNWER |
| KEKLVMKTHCPNDYQSAHYL | GVWKKPLCVGYERVVVKREL | KLSFPEGFNWERVMNFEDGG |
| HCPNDYQSAHYLNNDGKMAS | VGYERVVVKRELSAKPIQRV | NWERVMNFEDGGVVTVTQDS |
| AHYLNNDGKMASVKCPPKYE | KRELSAKPIQRVEPCTTCIT | EDGGVVTVTQDSSLQDGEFI |
| KMASVKCPPKYELTEDCNFC | IQRVEPCTTCITKCEPHGLV | TQDSSLQDGEFIYKVKLRGT |
| PKYELTEDCNFCRQMTGASL | TCITKCEPHGLVVRSTGFKI | GEFIYKVKLRGTNFPSDGPV |
| CNFCRQMTGASLKKGSYPLQ | HGLVVRSTGFKISSAVACAS | LRGTNFPSDGPVMQCRTMGW |
| GASLKKGSYPLQDLFCQSSE | GFKISSAVACASGVCVTGSQ | DGPVMQCRTMGWEASTERMY |
| YPLQDLFCQSSEDDGSKLKT | ACASGVCVTGSQSPSTEITL | TMGWEASTERMYPEDGALKG |
| QSSEDDGSKLKTKMKGVCEV | TGSQSPSTEITLKYPGISQS | ERMYPEDGALKGEIKQRLKL |
| KLKTKMKGVCEVGVQALKKC | EITLKYPGISQSSGGDIGVH | ALKGEIKQRLKLKDGGHYDA |
| VCEVGVQALKKCDGQLSTAH | ISQSSGGDIGVHMAHDDQSV | RLKLKDGGHYDAEVKTTYKA |
| LKKCDGQLSTAHEVVPFAVF | IGVHMAHDDQSVSSKIVAHC | HYDAEVKTTYKAKKPVQLPG |
| STAHEVVPFAVFKNSKKVYL | DQSVSSKIVAHCPPQDPCLV | TYKAKKPVQLPGAYNVDIKL |
| FAVFKNSKKVYLDKLDLKTE | VAHCPPQDPCLVHGCIVCAH | QLPGAYNVDIKLDILSHNED |
| KVYLDKLDLKTEENLLPDSF | PCLVHGCIVCAHGLINYQCH | DIKLDILSHNEDYTIVEQYE |
|  | CLVHGCIVCAHGLINYQCHT | HNEDYTIVEQYERAEGRHST |
|  |  | EQYERAEGRHSTGGMDELYK |
