# Supplementary material for: A DNA Vaccine Encoding the Gn Ectodomain of Rift Valley Fever Virus Protects Mice via a Humoral Response Decreased by DEC205 Targeting
Source: Front Immunol. 2019 Apr 25;10:860. doi: 10.3389/fimmu.2019.00860 (PMC6494931; doi:10.3389/fimmu.2019.00860)
Supplement: Supplementary file 3 [file Data_Sheet_3.docx]

Supplementary material 3. Relative RNA expression of peGn, pscDEC-eGn and pscCtrl-eGn in mouse skin. One hundred µg plasmids peGn (3 mice), pscDEC-eGn (4 mice) and pscCtrl-eGn (3 mice) were injected intradermally followed by EP. Twenty four hours later, RNA was extracted from skin biopsies. The eGn mRNA levels were measured using qRT-PCR, normalized with GAPDH and expressed as 2^- ΔCT^ values. Absence of residual plasmid in the RNA preparations was controlled using qPCR without reverse transcription.
